# Random allocation software for parallel group randomized trials

**DOI:** 10.1186/1471-2288-4-26

**Published:** 2004-11-09

**Authors:** Mahmood Saghaei

**Affiliations:** 1 Department of Anaesthesia, Isfahan University of Medical Sciences, Isfahan, Iran

## Abstract

**Background:**

Typically, randomization software should allow users to exert control over the different aspects of randomization including block design, provision of unique identifiers and control over the format and type of program output. While some of these characteristics have been addressed by available software, none of them have all of these capabilities integrated into one package. The main objective of the Random Allocation Software project was to enhance the user's control over different aspects of randomization in parallel group trials, including output type and format, structure and ordering of generated unique identifiers and enabling users to specify group names for more than two groups.

**Results:**

The program has different settings for: simple and blocked randomizations; length, format and ordering of generated unique identifiers; type and format of program output; and saving sessions for future use. A formatted random list generated by this program can be used directly (without further formatting) by the coordinator of the research team to prepare and encode different drugs or instruments necessary for the parallel group trial.

**Conclusions:**

Random Allocation Software enables users to control different attributes of the random allocation sequence and produce qualified lists for parallel group trials.

## Background

An important aspect of any trial that should be clearly stated in the final report is the method used to assign treatments (or other interventions) to participants [1]. In the final report of the trial, authors should specify the method of sequence generation, i.e. whether they have used mechanical means, a computer generated random list or random number table. After preparing a random sequence, subjects will be allocated to the trial groups using an implementation method such as numbered containers, central telephone line, or allocation by a person who is not involved in the main research and patient care (the encoder). During the process of allocation each subject will be given a unique identification code (Unique Identifier, UI). This UI will be used as a label to uniquely identify the patient's group after completion of the study. During the study period this UI will be given to the main researchers together with the necessary treatment (e.g. drug or placebo). Usually these treatments are prepared by the encoder with the same physical characteristics (shape, color, size, etc), differing only by the UI labels to blind the main researchers about the actual patient group. Following enrollment of all subjects into the study, these UIs are decoded to determine the patient group. Depending on the preference of the researchers or facilities of the research environment, subjects are randomly allocated to intervention groups using either a random list prepared before the study (In Advance method) or a randomized allocation at the moment of intended intervention (Just In Time method; JIT). Both the JIT and In Advance methods produce acceptable allocations, and the actual choice depends on the availability of certain facilities for each method. Usually the randomization components of these two methods are produced by running a randomization software on either an Internet service provider or a local computer. The local encoder will obtain the next allocation (JIT method) or the entire random list (In Advance method) from the service provider or from the software on local computer and prepare the necessary blinded equipments.

Without the use of computer software or Internet services the maintenance of the whole process of randomization and allocation is difficult. In addition, in the case of any necessary restrictions on the process of randomization (i.e. block randomization [2]) the complexity of the process will be increased even further and be prone to errors. Randomization software may run on a local computer or may be hosted by an Internet server. A complete list of these software and services can be found on Martin Bland's web site [3]. Most of randomization software are hosted by websites for both JIT and In Advance methods, which require access to the Internet [4-8]. However, most of these Internet services have restricted capabilities with respect to the block design specification, control over the output format and flexibility of UIs. Among these available services, the tool in Randomization.com [4] seems to be more advanced than the others. It allows users to specify the number of subjects per block, the number of blocks and up to 20 treatment labels. Therefore, this service produces simple and block randomization using fixed and equal block sizes. Unfortunately, this service does not allow further restriction on block design (e.g., multiple block lengths or random variation in block number or size). The generated random list is in the form of UI and group name pairs, formatted in a single column, which in cases of large sample sizes may require further work to fit it in multiple columns for fine printing. Moreover, the block borders are not visible to allow for easy visual inspection of block sizes and equality of cases. Although a minor problem, Randomization.com only produces sequential numeric UIs with variable lengths (e.g. 1, 10, 100). The variability in lengths of UIs may disturb the visual impact of the generated list compared to the fixed length UIs (e.g. 001, 010, 100). Some researchers prefer to use random UIs in mixed alphanumeric format to decrease the likelihood of memorization and to improve the blindness of the study and concealment of allocations. Available randomization software have more restrictions in their capabilities than the Internet randomization services. They have limitations in their output format [9] and users can not specify the number and naming of the treatment groups [10,11]. In addition, these software are not designed with the capability to produce flexible UIs for participants. Therefore, the main objective of Random Allocation Software was to construct a randomization software for parallel group trials with the following characteristics:

1. Independent running on a local computer without any need to access the Internet

2. Different types of program output: to file (html or text files), window and system clipboard

3. Provisions for different block design

4. Capability to deal with a larger number of groups

5. Specifying a name for each group

6. Control over the format, length and ordering of the generated UIs

7. Control over the format of generated sequence

8. Saving or loading the randomization settings

9. Viewing previously generated randomized sequences

## Implementation

Random Allocation Software is a program created in Microsoft Visual Basic 6, and it installs in the same way as ordinary Windows software (i.e. running setup.exe and following on screen instructions). Once installed and run, there are some controls in the main window for specifying the number of groups (2 to 16), sample size and the name of each group. It also contains menu items to determine the program output and randomization settings. The default program output is saved into either html or text files, and it may also have output to a window or to the system clipboard. A variety of randomization options can be set in the options window. The length of generated UIs (named as Code in the program) can be between 3 to 10 characters and there are options for different alphanumeric structures. In addition, these UIs can appear in sequential or random order in the generated random list. The program can generate simple or block randomization in different types, including equal size blocks, multiple block lengths with random variation among the specified block sizes and complete randomized blocks (random number and size of blocks). The generated sequence will appear in a multiple columns format and the number of columns (1 to 10) can be changed in the options window. Output to html file will be formatted in the form of one block per table. Borders of the tables may be shown or hidden. By clicking the 'Generate' button in the main window the random sequence will be generated and opened by the default viewer for the output file (e.g. Internet Explorer for html files). Previously created output files can also be viewed from inside the program. Additional options include saving the current randomization settings, loading a previous setting and enabling the program to save the last setting upon program exit.

During execution, the program produces a random sequence of allocation using the Rnd function that generates a floating point random number. The Rnd function uses the linear-congruent method for pseudo-random number generation as depicted by the following formula:

x1 = (x0 * a + c) MOD (2^24) [12]

where:

x1 = new value

x0 = previous value (an initial value of 327680 is used by Visual Basic unless the Randomize X function is used to specify a different seed as X)

a = 1140671485

c = 12820163

The seed of the Rnd will be the Timer function, which will return the number of seconds elapsed since midnight. Although this version of the program does not produce repeatable lists, it is possible to revise the program in subsequent versions to save the value of the seed to reproduce the same random list. The output consisted of shuffled allocations each of which is a UI, group name pair. The program checks for the uniqueness of the UIs and generates an error message if the specified UI length is insufficient to hold the entire sample size.

Runs test was used to check randomness of the output list with sample sizes from 10 to 190 (10, 30, 50, ..., 190) and from 200 to 3000 (200, 600, 1000, ..., 3000). Each runs test was carried out for the group number of 2, 3, 5 and 6. SPSS 10 software was used to perform the runs test.

## Results

**Figure 1:**
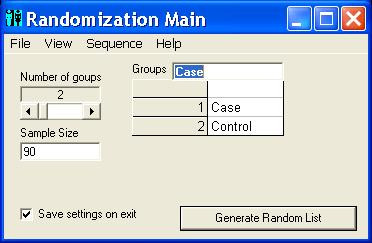
**Main window. **The main window showing different options for number of groups, sample size, and group names.

**Figure 2:**
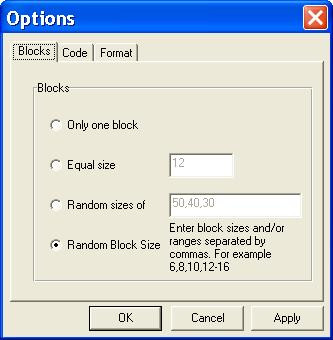
**Options window: Blocks. **Options window, settings for block design.

**Figure 3:**
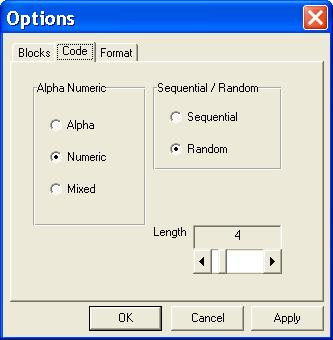
**Options window: Code. **Options window, setting the format of unique identifier (UI) specified in the program as Code.

The program starts running with the default settings. Users may run the program with the default settings or set the number of groups, the name of each group and the sample size. Clicking the 'Generate' button (figure 1) produces the random sequence. Before generating the random sequence, the option window will be displayed and different randomization settings can be entered (figure 2 and 3). Consider, for example, that we want to produce a simple randomized list for a sample size of 30 subjects into three groups of Case, Control and Placebo with numeric sequential UIs. After setting different options and clicking the 'Generate' button, the generated list will appear in columns (Table 1). Each entry in the list consists of a UI, and a group name pair. Alternatively numeric UIs may appear in random order (Table 2). Table 3 shows the output of the program for a block randomization with blocks of equal sizes.

**Table 1:** A simple randomized list produced by the software for a sample size of 30 subjects into three groups of Case, Control and Placebo with numeric sequential unique identifiers

001: Case	009: Placebo	017: Control	025: Placebo
002: Control	010: Control	018: Control	026: Case
003: Case	011: Placebo	019: Case	027: Case
004: Case	012: Control	020: Control	028: Placebo
005: Control	013: Case	021: Placebo	029: Placebo
006: Placebo	014: Case	022: Case	030: Control
007: Placebo	015: Placebo	023: Case	
008: Control	016: Control	024: Placebo	

**Table 2:** The same setting as in table 1, but with the numeric UIs in random order

288: Case	200: Control	462: Placebo	775: Case
644: Control	437: Case	448: Case	622: Control
278: Placebo	364: Control	523: Control	327: Control
427: Case	525: Control	837: Case	514: Placebo
146: Placebo	796: Case	804: Placebo	610: Case
383: Placebo	208: Control	581: Control	167: Placebo
493: Placebo	862: Placebo	181: Control	
484: Case	079: Case	254: Placebo	

**Table 3:** A block randomization list with four blocks of equal sizes

504: Placebo	671: Placebo	767: Case	442: Control	094: Placebo
256: Case	636: Case	200: Control	677: Control	
669: Placebo	355: Control	334: Case	765: Control	073: Control
377: Case	537: Placebo	527: Placebo	485: Case	
183: Placebo	658: Control	612: Case	875: Control	888: Case
733: Case	127: Placebo	864: Placebo	476: Control	
552: Control	138: Case	548: Case	938: Placebo	592: Placebo
810: Control	213: Control	584: Placebo	438: Case	

**Figure 4:**
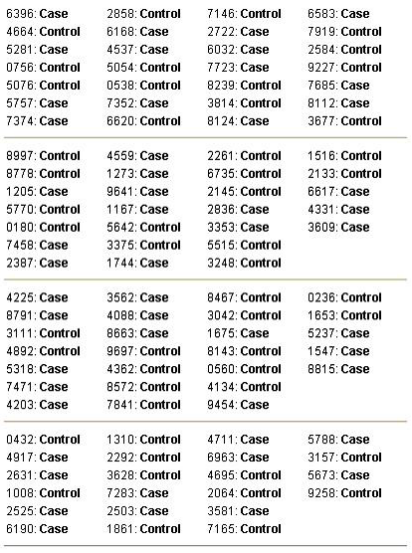
**Sample output. **Sample output for a block randomization with random block sizes.

Figure 4 is the printed output of the program for a block randomization with random block sizes.

In block randomization the final sample size is usually larger than the specified one.

A total of 18 runs test were performed to check the randomness of the program output, which resulted in P values of 0.22 to 0.81.

## Discussion

The main use of Random Allocation Software is to produce simple or block randomized sequences for parallel group trials. Its use is restricted to parallel group randomized trials. Compared with similar software, it enables the user to control the length, order and format of the UIs; and the type and format of the output. It allows specifying up to 16 groups for parallel trials.

## Conclusions

Random Allocation Software has been designed to produce random sequences consisting of UI, group name pairs with additional control over the output format and type. Available randomization software generally has limitations in the number of groups, naming each group, generating UIs and control over the output. Many of these problems have been addressed in the present software. As has been stated in previous sections, the main use of this software is for randomization in parallel group trials. The software can be revised to support crossover and other types of randomized trials. The experienced user may test the randomness of the program output by selecting numeric labels for group names and then exporting the generated list into a statistical software such as SPSS to execute a runs test on the exported data.

## Availability and requirements

Project name: Random Allocation Software

Public use access:

 (Latest version)



Operating systems: Windows 98, Me, 2000, XP. It should be noted that on some Windows operating systems (especially Windows 2000) during installation of the program an error message like "Setup Cannot Continue... System Files Are Out of Date" may be displayed. If this happens, click OK and restart the system. Then run the setup.exe again. This is due to a known bug in the installation programs of Microsoft Visual Basic [13]. This problem has been removed from the newer versions of the program. Users are recommended to download the latest version from the first address.

Programming Language: Visual Basic 6

Other requirements: Internet Browser (Internet Explorer 5 or higher is recommended)

License: Free for academic use.

## Abbreviations

JIT = Just in Time

UI = Unique Identifier

## Competing interests

The author declares that he has no competing interests.

## Pre-publication history

The pre-publication history for this paper can be accessed here:


